# Recasting the (Synchrosqueezed) Short-Time Fourier Transform as an Instantaneous Spectrum

**DOI:** 10.3390/e24040518

**Published:** 2022-04-06

**Authors:** Steven Sandoval, Phillip L. De Leon

**Affiliations:** Klipsch School of Electrical and Computer Engineering, New Mexico State University, Las Cruces, NM 88003, USA; pdeleon@nmsu.edu

**Keywords:** frequency-domain analysis, signal analysis, signal representation, spectral analysis

## Abstract

In a previous work, we proposed a time-frequency analysis called instantaneous spectral analysis (ISA), which generalizes the notion of the Fourier spectrum and in which instantaneous frequency is utilized to the fullest extent. In this paper, we recast both the Fourier transform (FT) and filterbank (FB) interpretations of the short-time Fourier transform (STFT) as instantaneous spectra. We show that to recast the FB interpretation of STFT as an instantaneous spectrum with valid structure, frequency reassignment is a fundamental necessity, thus demonstrating that this IS is closely related to the synchrosqueezed STFT. This result provides a new theoretical motivation for the synchrosqueezed STFT. Finally, we illustrate through example the instantaneous spectra corresponding to the FT and FB interpretations of STFT using two closed-form examples.

## 1. Introduction

In Gabor’s seminal work [1], the notion of joint time-frequency analysis was proposed and led to the development of the short-time Fourier transform (STFT) [2,3]. Even today, STFT is the most well-known and utilized method in time frequency analysis [4,5,6,7,8,9] and extensions such as the synchrosqueezing transform (SST) and synchrosqueezed STFT are still actively being investigated [10]. Synchrosqueezing and reassignment are typically motivated as post-processing techniques in order to improve readability in the time-frequency plane [11,12] or as refinements based on phase information.

In [13], we developed a generalized framework for time-frequency analysis which we termed instantaneous spectral analysis (ISA) and proved that the instantaneous spectrum (IS) exactly localizes signal components in an instantaneous bandwidth sense. Using ISA, we are able to define more general time-frequency spectra than is possible using STFT. This is a result of the basic time-frequency atom or component utilized in these methodologies. More specifically, ISA theory allows the use of an AM–FM component whereas STFT uses a far more restrictive component. Moreover, IS theory allows the unambiguous specification of an IS S(t,ω) and a related complex-valued signal z(t) corresponding to a component set S

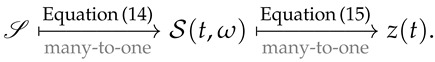

Although this may be considered as an *ideal synthesis model* for instantaneous spectra and AM–FM models, the many-to-one mappings are sources of information loss which allow an infinite number of instantaneous spectra and component sets to map to the same signal. As a result, the reverse process is under determined and *no unique analysis model exists*.

One approach which can be taken for the analysis stage is to consider two stages: (1) signal decomposition and (2) component demodulation

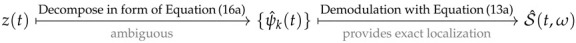

where all ambiguity lies in how the signal z(t) is decomposed into a set of components—there exist an infinite number of ways to express a whole as a sum of parts. However, every decomposition has the advantage that it can be associated with an instantaneous spectrum in which each component is exactly localized. Thus, ISA can be immediately used to enhance existing decomposition methods with an associated instantaneous spectrum with exact time-frequency localization. However, while the distinct separation of the analysis stage into decomposition and demodulation is well suited for pairing ISA with decomposition methods and AM–FM models, alternate approaches to the analysis stage exist.

For example, there exists a great body of literature devoted to the study of time-frequency distributions (TFDs), and although TFDs and ISs are both mathematical objects which describe signals in time-frequency spaces, they describe different types of spaces. Thus, *it is natural to seek to establish the connection of particular TFDs as special cases of ISs, if and when this is possible*. Let T denote an integral transformation relating a complex-valued signal z(t) and a TFD Z(t,ω), and $T^{-1}$ denote its inverse, assuming it exists

$$\begin{array}{c} z\left(t\right)\overset{T}{\underset{T^{-1}}{⇌}}Z\left(t,\omega \right). \end{array}$$

The focus of traditional TFD analysis may be considered as the theory regarding the existence and mathematical properties for different choices of the transformation T. ISA theory can be used in conjunction with other time-frequency methods as a framework to pose and address other important questions. For example, suppose we wish to study the performance of a particular integral transformation T on a particular class of signals. If we represent the particular class of signals by constraints on the form of the components in S, then we can compare the IS obtained by recasting the time-frequency distribution Z(t,ω), denoted by $\hat{S}\left(t,\omega \right)$, by considering

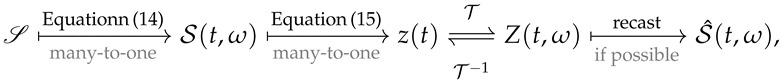

and comparing $\hat{S}\left(t,\omega \right)$ to S(t,ω).

The purpose of this work is to show how to enforce the structure necessary to recast the time-frequency distribution Z(t,ω) to an instantaneous spectrum $\hat{S}\left(t,\omega \right)$, if we choose the integral transformation T as the STFT. We show that there are two ways to do the recasting, each corresponding to one of the two classic interpretations of STFT. Our contributions are as follows:

1. We show that the two equivalent STFT interpretations lead to different ISs, and thus provide new insights into STFT. In particular, we show that the IS corresponding to the FT interpretation of STFT corresponds to an IS for each window grain, while on the other hand, a single IS corresponding to the FB interpretation of STFT exists when STFT is synchrosqueezed [14,15]. As a result, in the FT interpretation, the components have a restrictive fixed amplitude and fixed frequency, while in the FB interpretation, the components are AM–FM in nature. This results in significant conceptual and practical differences between the two interpretations.
2. We contribute a new theoretical motivation for synchrosqueezing. In particular, in order to recast the FB interpretation as an IS, we show that reassignment in frequency is a fundamental requirement. This is in contrast to the view of synchrosqueezing largely as a heuristic approach to improve energy concentration in the time-frequency plane.
3. By recasting the two STFT interpretations as an IS, we can leverage the 3D IS visualization [13] to contribute a novel visualization of STFT. This is advantageous because the 3D IS allows visualization of multiple aspects of the signal decomposition simultaneously, including both magnitude and phase of each signal component. While it may take the reader some time to become comfortable with the 3D visualization, we believe it has significant advantages in terms of interpretability and note that the STFT phase spectrum is almost never considered or visualized.

For the benefit of the reader, this paper reviews key concepts of STFT and synchrosqueezed STFT, however, this work is not intended to be a review paper. For such reviews, we refer the reader to [2,3] for STFT and [14,16] for reassignment and synchrosqueezing. Rather, this paper recasts STFT as an IS, which we believe to be a more powerful signal analysis framework. The remainder of this paper is organized as follows. In Section 2, we introduce our notation for the continuous STFT and provide the expressions specifically related to the FT and FB interpretations. In Section 3, we provide a brief history of the development of reassignment techniques leading to the synchrosqueezed STFT. In Section 4, we provide a brief summary of ISA theory. However, as this work is an extension of our prior work, it is strongly recommended that [13] be read in advance. In Section 5, we give our main contribution by recasting the FT and FB interpretations of STFT as an IS. In Section 6, we provide illustrations and discussion on the relationships of the FT and FB interpretations of STFT to IS for example signals. Finally, in Section 7, we provide concluding remarks.

## 2. The Short-Time Fourier Transform

In this section, we review the (continuous) STFT following the development of the discrete STFT by Allen and Rabiner [2]. We begin by choosing a real, even-symmetric window function w(·) such that

(1) $$\int_{-\infty }^{\infty }w\left(\tau \right)\;d\tau =1.$$

This ensures that

(2) w(t−τ)=w(τ−t)

and superimposing all “window grains” z(t)w(τ−t) over τ gives

(3) $$z\left(t\right)=\int_{-\infty }^{\infty }z\left(t\right)w\left(\tau -t\right)\;d\tau .$$

Next, we review the two interpretations of STFT [2,3].

### 2.1. Fourier Transform Interpretation of STFT

The FT of (3) yields

(4a) $$\;\;\;\;\;Z(j\omega )=\int_{-\infty }^{\infty }\left[\int_{-\infty }^{\infty }z\left(t\right)w\left(\tau -t\right)\;d\tau \right]e^{-j\omega t}\;dt$$

(4b) $$\begin{array}{ccc} & \;\;\;\;\;\;\;\;\;\;= & \int_{-\infty }^{\infty }\left[\int_{-\infty }^{\infty }z\left(t\right)w\left(\tau -t\right)e^{-j\omega t}\;dt\right]\;d\tau \end{array}$$

(4c)    =∫−∞∞Fz(t)w(τ−t)00dτ

(4d) $$\begin{array}{ccc} & = & \int_{-\infty }^{\infty }Z_{w}\left(\omega ;\tau \right)\;d\tau \end{array}$$

where $Z_{w}\left(\omega ;\tau \right)$ denotes the classical (the use of *classical* and *modified* for describing STFT is based on the current literature). See for example [4,17,18,19]) STFT. Equating the expressions inside the integrals of (4c) and (4d), shows that STFT may be considered as the FT of all window grains

(5) Zw(ω;τ)≜Fz(t)w(τ−t)00.

This may be viewed as a function of ω at a fixed value of time shift τ. The signal may be recovered by means of the overlap-add (OLA) method for short-time synthesis

(6) z(t)≜∫−∞∞F−1Zw(ω;τ)00dτ.

### 2.2. Filterbank Interpretation of STFT

The FB interpretation of the modified STFT may be developed by considering $w\left(t\right)e^{j\nu t}$ as a channelizer with center frequency ν

(7) $$Z_{w}\left(t;\nu \right)=z\left(t\right)*w\left(t\right)e^{\;j\nu t}$$

where ∗ denotes convolution. Equivalently, the classical STFT

(8) Zw(t;ν)e−jνt≜z(t)e−jνt00∗w(t)

which may be viewed as a function of *t* at a fixed value of ν, i.e., the signal is frequency shifted and filtered with the impulse response w(t) on a continuum of frequency shifts ν. The signal may be recovered by means of the filterbank summation (FBS) method for short-time synthesis

(9) $$\begin{array}{ccc} z(t) & ≜ & \frac{1}{2\pi w(0)}\int_{-\infty }^{\infty }Z_{w}\left(t;\nu \right)\;d\nu \\ \; & = & \frac{1}{2\pi w(0)}\int_{-\infty }^{\infty }\left[z\left(t\right)*w\left(t\right)e^{\;j\nu t}\right]\;d\nu \\ \; & = & \frac{1}{2\pi w(0)}\int_{-\infty }^{\infty }z\left(\tau \right)w\left(t-\tau \right)\left[\int_{-\infty }^{\infty }e^{\;j\nu (t-\tau )}\;d\nu \right]\;d\tau \\ \; & = & \frac{1}{2\pi w(0)}\int_{-\infty }^{\infty }z\left(\tau \right)w\left(t-\tau \right)2\pi \delta \left(t-\tau \right)\;d\tau \\ \; & = & \frac{1}{w(0)}z\left(t\right)w\left(0\right). \end{array}$$

### 2.3. Complimentary Interpretations

We point out to the reader a crucial difference between the meaning of the independent variables in (5) and (8) even though these equations are equivalent

(10) $$Z_{w}\left(t;\nu \right)e^{-j\nu t}\equiv Z_{w}\left(\omega ;\tau \right).$$

In (5), τ is a time shift variable and ω is instantaneous frequency (IF), while in (8), *t* is time instant and ν is a frequency shift variable. While this difference is well known and insignificant in STFT theory, it results in major differences in the relationship of STFT to IS based on the interpretation taken. Note that regardless of how STFT is computed, we may switch interpretations by interchanging variables t↔τ and variables ω↔ν in (10).

## 3. Synchrosqueezed Short-Time Fourier Transform

In the 1970s, Kodera et al. [11,20] proposed to modify the spectrogram by taking into account the phase information that is usually discarded. The basic idea is to reassign energy in the spectrogram to a new time and frequency location by utilizing phase derivatives. Kodera’s work received little attention for decades [14], and in the 1980s Friedman also proposed spectrogram reassignment [21], apparently without knowledge of the work by Kodera. In Friedman’s approach, reassignment occurs in frequency but not in time. In both approaches, phase is used to perform reassignment but is subsequently discarded, thus preventing reconstruction. Slow adoption of these methods is often attributed to the inability to reconstruct the signal and the numerical problems associated with derivative approximations [12,14,16]. Auger and Flandrin showed that the numerical problems associated with the phase derivative could be avoided by computing three STFTs with related window functions, which led to a more efficient implementation.

In the 1990s, reassignment resurfaced when two independent groups developed the reassignment method (RM) [12,14,16] and the SST [22,23]. The RM developed by Auger and Flandrin is similar to Kodera’s work in that reassignments occur in both time and frequency. Additionally, they showed that the reassignment concept could be generalized to work for a broader class of time-frequency representations, e.g.,  in the Wigner–Ville distribution by recasting the problem in terms of centroids instead of phase derivatives. The SST developed by Maes and Daubechies is similar to Friedman’s work in that reassignments occur only in frequency using phase derivatives. However, differences from Friedman’s work include the use of a complex wavelet transform instead of the STFT and a reassignment of a complex value rather than a real value.

In [14,24], the SST is computed using an STFT rather than a wavelet transform, leading to the synchrosqueezed STFT

SST(t,ω)=12πw(0)∫−∞∞Zw(t;ν)ejωtδω−00ω^(t;ν)00  dν

where

$$\begin{array}{c} \hat{\omega }\left(t;\nu \right)=\frac{\;d}{\;dt}\arg\left\{Z_{w}\left(t;\nu \right)\right\}. \end{array}$$

## 4. Instantaneous Spectral Analysis

In [13], we introduced ISA as a general framework for time-frequency analysis consisting of three parts: (1) a parameter set, (2) an IS, and (3) a complex AM–FM signal model. More specifically, in this framework: (1) a signal is represented by a set of canonical triplets $S≜\left\{C_{0},C_{1},\ldots ,C_{K-1}\right\}$, (2) each set has a single-valued mapping to an IS S↦S(t,ω), and (3) each IS has a single-valued mapping to a signal S(t,ω)↦z(t)

(11) $$S=\left\{C_{k}\right\}\overset{\mathrm{Equation}\;(14)}{⟼}S\left(t,\omega \right)\overset{\mathrm{Equation}\;(15)}{⟼}z\left(t\right).$$

The canonical triplet for the *k*th AM–FM component is

(12) Ck≜ak(t),ωk(t),ϕk00

where $a_{k}\left(t\right)$ is the instantaneous amplitude (IA), $\omega_{k}\left(t\right)$ is the IF, and $\phi_{k}$ is the phase reference. The *k*th complex AM–FM component is then given by

(13a) ψkt;Ck00≜ak(t)ej∫−∞tωk(τ)  dτ+ϕk

(13b) $$\begin{array}{ccc} & = & a_{k}\left(t\right)e^{\;j\theta_{k}\left(t\right)} \end{array}$$

(13c) $$\begin{array}{ccc} & \;= & s_{k}\left(t\right)+j\sigma_{k}\left(t\right) \end{array}$$

where $\theta_{k}\left(t\right)$ is the phase function, $s_{k}\left(t\right)$ is the real part, and $\sigma_{k}\left(t\right)$ is the imaginary part. With (12) and (13), the IS is defined as

(14) S(t,ω;S)≜2π∑k=0K−1∫−∞∞ψkτ;Ck002δt−τ,00ω−ωk(τ)00 dτ=2π∑k=0K−1ψkt;Ck00δ00ω−ωk(t)00.

The IS S(t,ω;S) maps to the complex signal z(t;S) with

(15) $$\frac{1}{2\pi }\int_{-\infty }^{\infty }S\left(t,\omega ;S\right)\;d\omega =z\left(t;S\right).$$

Finally, the complex signal z(t;S) is represented as a superposition of *K* (possibly infinite) complex AM–FM components

(16a) zt;S00≜∑k=0K−1ψkt;Ck00

(16b) $$\begin{array}{ccc} & \;\;\;= & x(t)+jy(t). \end{array}$$

We refer the reader to [13] for additional details.

We emphasize to the reader that although IS is expressed by a (complex-valued) function of *t* and ω, not every function of *t* and ω has the necessary structural requirements to be a valid IS. This is not unlike STFT which is also a (complex-valued) function of time and frequency and where not every function of time and frequency has the necessary structural requirements to be a valid STFT. For example, it is well understood that when modifying the STFT magnitude there is the distinct possibility that modification may lead to an invalid STFT. In this case, inversion requires algorithms such as least-squared error inverse STFT (LSE-ISTFT) which inverts the invalid STFT to the signal which has an STFT closest (in an LSE sense) to the invalid STFT [25,26,27].

Moreover, although both the STFT and IS provide spectral representations in time and frequency, they have different structural requirements. This is due to the fact that the requirements are imposed by the analysis equations in (5) and (8), whereas the requirements of IS are imposed by the definition in (14). One implication of this is that one cannot assume that an STFT has the necessary structure to be a valid IS. On one hand, we show that although the FT interpretation of STFT does not possess the necessary structure to be a valid IS, it may be interpreted as a continuum of ISs. On the other hand, we show that while the FB interpretation of STFT does not possess the necessary structure to be a valid IS, the structure necessary to utilize ISA theory may be imposed by synchrosqueezing the STFT.

### Relation to Frequency Domain Analysis

In [13], we gave proof that frequency domain analysis corresponds to a *specialized* (and restricted) form of an IS when $a_{k}\left(t\right)=a_{k}\omega_{0}$, $\omega_{k}\left(t\right)=k\omega_{0}$, and the discrete set takes on a continuum, i.e., $\omega_{0}\to 0$,

(17) Z(jω)ejωt=limω0→0S(t,ω)000ak(t)=akω0ωk(t)=kω0=SFD(t,ω).

Finally, evaluating (17) at t=0 yields

(18) $$Z\left(j\omega \right)=S^{\mathrm{FD}}\left(0,\omega \right).$$

We refer the reader to [13] for additional details.

## 5. Recasting the Short-Time Fourier Transform as an IS

IS provides a signal analysis which is both instantaneous in *t* and ω. From Section 2, we see that STFT allows for instantaneous analysis in only one of the variables, i.e., the FB interpretation is instantaneous in time while the FT interpretation is instantaneous in frequency (albeit constant frequency). In this section, we recast each STFT interpretation as an IS. While the two interpretations are conceptual in nature, the ISs corresponding to these interpretations take on different mathematical forms. As we show, the IS corresponding to the FB interpretation $S^{\mathrm{FB}}\left(t,\omega \right)$ makes use of AM–FM components, and thus is easily understood in terms of a single IS. On the other hand, the IS corresponding to the FT interpretation $S_{\tau }^{\mathrm{FT}}\left(t,\omega \right)$ uses a more restrictive component and is best understood using a continuum of ISs. In this section, we continue from (11) and develop (19), (20), and (24), whose context in the overall theory is illustrated below

$$\begin{array}{ccccccc} z(t)\;\; & \overset{F}{⟼} & \;\;Z(j\omega ) & \;\;\overset{\mathrm{Equation}\;(17)}{⟼} & S^{\mathrm{FD}}\left(t,\omega \right)\\ z\left(t\right)\;\; & \overset{\mathrm{Equation}\;(5)}{⟼} & Z_{w}\left(\omega ;\tau \right) & \;\;\overset{\mathrm{Equation}\;(19)}{⟼} & S_{\tau }^{\mathrm{FT}}\left(t,\omega \right) & \;\;\overset{\mathrm{Equation}\;(20)}{⟼} & S^{\mathrm{FD}}\left(t,\omega \right).\\ z(t)\;\; & \overset{\mathrm{Equation}\;(7)}{⟼} & Z_{w}\left(t;v\right) & \;\;\overset{\mathrm{Equation}\;(24)}{⟼} & S^{\mathrm{FB}}\left(t,\omega \right) \end{array}$$

### 5.1. IS Corresponding to the FT Interpretation of STFT

The FT interpretation of the classical STFT in (5) is that of a continuum of FTs indexed by τ. Thus, from the relation of the FT to the IS in (17), the IS corresponding to the window grain at t=τ is

(19) $$S_{\tau }^{\mathrm{FT}}\left(t,\omega \right)=Z_{w}\left(\omega ;\tau \right)e^{\;j\omega t}.$$

Superimposing the ISs in (19) gives the IS corresponding to the FT interpretation of STFT

(20) $$S^{\mathrm{FD}}\left(t,\omega \right)=\int_{-\infty }^{\infty }S_{\tau }^{\mathrm{FT}}\left(t,\omega \right)\;d\tau .$$

Equation (4d) shows that STFT is a decomposition of FT. Likewise, the continuum of ISs corresponding to the window grains decomposes the IS corresponding to the FT interpretation

(21a) $$\begin{array}{ccc} \int_{-\infty }^{\infty }S_{\tau }^{\mathrm{FD}}\left(t,\omega \right)\;d\tau & = & \int_{-\infty }^{\infty }Z_{w}\left(\omega ;\tau \right)e^{\;j\omega t}\;d\tau \end{array}$$

(21b) $$\begin{array}{cc} & \;\;\;\;\;\;=e^{\;j\omega t}\int_{-\infty }^{\infty }Z_{w}\left(\omega ;\tau \right)\;d\tau \end{array}$$

(21c) $$\begin{array}{ccc} & \;\;\;= & e^{\;j\omega t}Z\left(j\omega \right) \end{array}$$

(21d) $$\begin{array}{ccc} & \;\;\;= & S^{\mathrm{FD}}\left(t,\omega \right). \end{array}$$

In other words, superimposing the ISs corresponding to the FT interpretation of STFT yields the IS corresponding to frequency domain analysis (Fourier transform) $S^{\mathrm{FD}}\left(t,\omega \right)$, and as a result does not provide a new IS to study. Rather, when taking the FT interpretation of STFT, we only gain new insights by studying the ISs corresponding to the window grains.

### 5.2. IS Corresponding to the FB Interpretation of STFT

The FB interpretation of the modified STFT in (7) is that of an infinite number of signal components, each corresponding to frequency shift −ν followed by filtering (convolution) with w(·). Naively comparing (15) with (9) one might be tempted to assume that a corresponding IS may be formed with

(22) SFB(t,ω)=1w(0)Zw(t;ν)00ν→ω.

However, this would be incorrect because it does not provide the structure necessary to be a valid IS. This is further illustrated and discussed below. On the other hand, we can construct an IS with valid structure from $Z_{w}\left(t;\nu \right)$ by reassigning the component associated with frequency shift ν to the appropriate IF. We begin by writing the modified STFT in polar form as

(23) Zw(t;ν)=aw(t;ν)expjθw(t;ν)00.

Using the IS definition in (14) we have

(24) SFB(t,ω)=∫−∞∞aw(t;ν)ejθw(t;ν)δω−00ddtθw(t;ν) dν

which is immediately recognized as a synchrosqueezed STFT [14,15]. We note that while most developments of reassignment/synchrosqueezing are motivated as post-processing techniques in order to improve readability of spectrograms, in our development, reassignment in frequency is a fundamental necessity to ensure a valid IS structure. Furthermore, techniques which reassign in time are not compatible with a valid IS structure.

### 5.3. Discussion

A critical difference between the ISs corresponding to FT and FB interpretations of STFT is the form of the components utilized. With the FT interpretation, the individual components are obtained from the classical STFT $Z_{w}\left(\omega ;\tau \right)$, as follows. For each point (τ,ω), the component is formed as

(25) $$\psi_{\tau ,\omega }\left(t\right)=Z_{w}\left(\omega ;\tau \right)e^{\;j\omega t}.$$

Here, $Z_{w}\left(\omega ;\tau \right)$ acts as an initial condition, and multiplication with $e^{\;j\omega t}$ projects the component forward and backward in time. With the FB interpretation, the individual components are obtained from the modified STFT $Z_{w}\left(t;\nu \right)$, as follows. For each frequency shift ν, the component is formed as

(26) $$\psi_{\nu }\left(t\right)=Z_{w}\left(t;\nu \right).$$

While (25) and (26) have similar mathematical forms, they are very different because $Z_{w}\left(\omega ;\tau \right)$ is independent of *t* while $Z_{w}\left(t;\nu \right)$ is dependent on *t*. As a result, the components in (25) have a fixed amplitude $|Z_{w}\left(\omega ;\tau \right)|$ and fixed frequency ω, while the components in (26) have, in general, a time-varying amplitude $|Z_{w}\left(t;\nu \right)|$ and time-varying frequency $\frac{\;d}{\;dt}\arg\left\{Z_{w}\left(t;\nu \right)\right\}$. Thus, the former component is very restrictive, whereas the latter component is AM–FM in nature. This results in significant conceptual and practical differences between $S_{\tau }^{\mathrm{FT}}\left(t,\omega \right)$ in (19) and $S^{\mathrm{FB}}\left(t,\omega \right)$ in (24), even though mathematically there is little practical difference between $Z_{w}\left(\omega ;\tau \right)$ and $Z_{w}\left(t;\nu \right)$. Moreover, (26) along with (23) explains why $Z_{w}\left(t;\nu \right)$ does not have the necessary structure to be interpreted as an IS: the energy associated with the component $\psi_{\nu_{0}}\left(t\right)$ is located at channelizer frequency $\nu =\nu_{0}$ in $Z_{w}\left(t;\nu_{0}\right)$, rather than at the appropriate IF location $\frac{\;d}{\;dt}\arg\left\{Z_{w}\left(t;\nu_{0}\right)\right)\}$.

## 6. Instantaneous Spectra Corresponding to STFT Interpretations for Example Signals

In this section, we illustrate through example the IS corresponding to the two interpretations of STFT. Information regarding visualization of the IS can be found in [13] and software for IS visualization at [28,29]. The examples shown below consist of two signals which can be expressed and analyzed in a closed-form as well as a real world signal, i.e., acoustic recording. In order to develop closed-form expressions for STFT, we choose to analyze the complex exponential and linear FM chirp with a Gaussian window. Our analysis uses the following FT pairs. First, the FT of a quadratic chirplet is given by

(27) $$\begin{array}{c} z\left(t\right)=\exp\left(-p_{1}t^{2}\right)\\ ↕F\\ Z\left(j\omega \right)=\sqrt{\frac{\pi }{p_{1}}}\exp\left[-\frac{1}{4p_{1}}\omega^{2}\right] \end{array}$$

where $p_{1}\in C$ and Re{p1}>0. Second, it can be shown by completing the square and using (27), that the FT of a product of time-shifted quadratic chirplets is given by

(28) $$\begin{array}{c} z\left(t\right)=\sqrt{\frac{p_{2}}{\pi }}\exp\left[-p_{1}{\left(t-t_{1}\right)}^{2}\right]\exp\left[-p_{2}{\left(t-t_{2}\right)}^{2}\right]\\ ↕F\\ Z\left(j\omega \right)=c\;\exp\left(-\frac{1}{4p_{3}}\omega^{2}\right)\exp\left(-jT\omega \right) \end{array}$$

with the chirp parameters $p_{1}\in C$, $p_{2}\in C$, Re{p1}>0, Re{p2}>0, $p_{3}=p_{1}+p_{2}$, $T=\left(t_{1}p_{1}+t_{2}p_{2}\right)/p_{3}$, and $c=\sqrt{p_{2}/p_{3}}\exp\left[-(t_{1}^{2}p_{1}+t_{2}^{2}p_{2}-T^{2}p_{3})\right]$.

### 6.1. Complex Exponential

In the first example, consider the canonical triplet

(29) $$C_{0}=\left(1,\;\;\omega_{0},\;\;0\right)$$

which using (16a) gives the complex exponential signal

(30) $$z\left(t\right)=\exp\left(j\omega_{0}t\right)$$

and with (14) gives the IS

(31) $$S\left(t,\omega \right)=2\pi \delta \left(\omega -\omega_{0}\right)e^{\;j\omega t}.$$

Choosing a Gaussian window

(32) $$w\left(t\right)=\sqrt{\frac{\beta^{2}}{2\pi }}\exp\left[-\frac{\beta^{2}t^{2}}{2}\right]$$

we compute the STFT corresponding to the FT interpretation by using (5), choosing $p_{1}=\beta^{2}/2$ in (27), and using time- and frequency-shift properties of the FT

(33) Zw(ω;τ)=exp−(ω−ω0)22β2−jτ(ω−ω0)00.

With (33) and (10) we then form the IS corresponding to the FT interpretation

(34) SτFT(t,ω)=exp−(ω−ω0)22β2−jτ(ω−ω0)00ejωt.

Next, we compute the STFT corresponding to the FB interpretation using (33) and (10)

(35) Zw(t;ν)=exp−(ν−ω0)22β2+jθw(t;ν)00

where

(36) $$\theta_{w}\left(t;\nu \right)=\omega_{0}t.$$

Substituting into (24), we form the IS corresponding to the FB interpretation

(37) SFB(t,ω)=∫−∞∞exp−(ν−ω0)22β2ejtω0δω−00ω0 dν=β2πδω−00ω000ejω0t.

Comparing (37) with (31), we see that after reassignment $S^{\mathrm{FB}}\left(t,\omega \right)$ yields the correct IS (to within a scale factor) for this signal. On the other hand, direct comparison of (34) with (31) is not possible. While one could superimpose (34) on the continuum τ as described in (20), this would only lead to $S^{\mathrm{FD}}\left(t,\omega \right)$. Although this does lead to a valid IS, it is not useful for time-frequency analysis because it provides the same information as FT.

**Figure 1:**
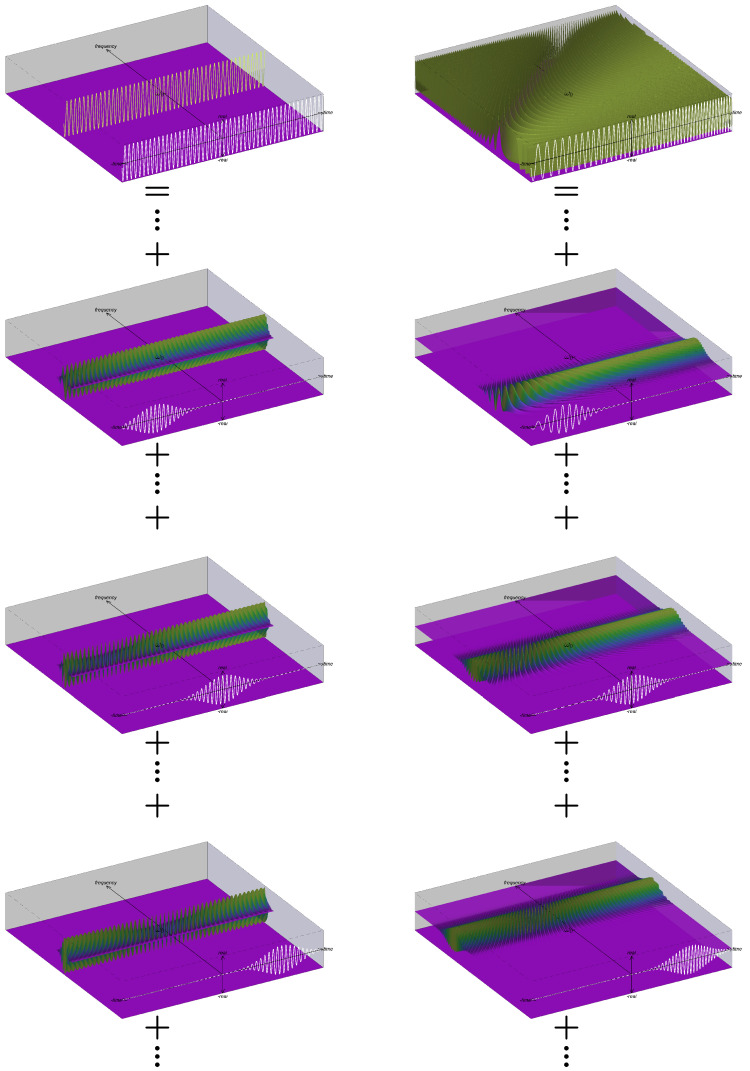
Illustration of the instantaneous spectra associated with Fourier transform interpretation of the STFT for a (**left column**) complex exponential and (**right column**) linear FM chirp. For each of the signals, the top plot shows $S^{\mathrm{FT}}\left(t,\omega \right)$ while the lower three plots show $S_{\tau }^{\mathrm{FT}}\left(t,\omega \right)$ for three instances of τ. The superposition of the ISs of the window grains yields the IS of the FT and is visually demonstrated by the “+” notation used in the figure.

The ISs corresponding to the FT interpretation of the STFT in (34) are shown in the left column of Figure 1. The top plot shows $S^{\mathrm{FD}}\left(t,\omega \right)$ for the complex exponential while the lower three plots show $S_{\tau }^{\mathrm{FT}}\left(t,\omega \right)$ for three different τ [see (19)]. The fact that the FT interpretation yields a representation that may be considered as a decomposition into a continuum of ISs of the window grains at each τ [see (21d)] is visually demonstrated by the “+” notation used in the figure. From the figure, it is apparent that the frequency spectrum which results from taking the FT of any window grain has components that extend beyond the time support of the window. Thus, even if a window grain has finite time support, the associated frequency spectrum has infinite extent and is not simply limited to the local vicinity of the window. This demonstrates that while the STFT is mathematically correct, there is a conceptual flaw in the IS obtained by recasting the FT interpretation of STFT.

**Figure 2:**
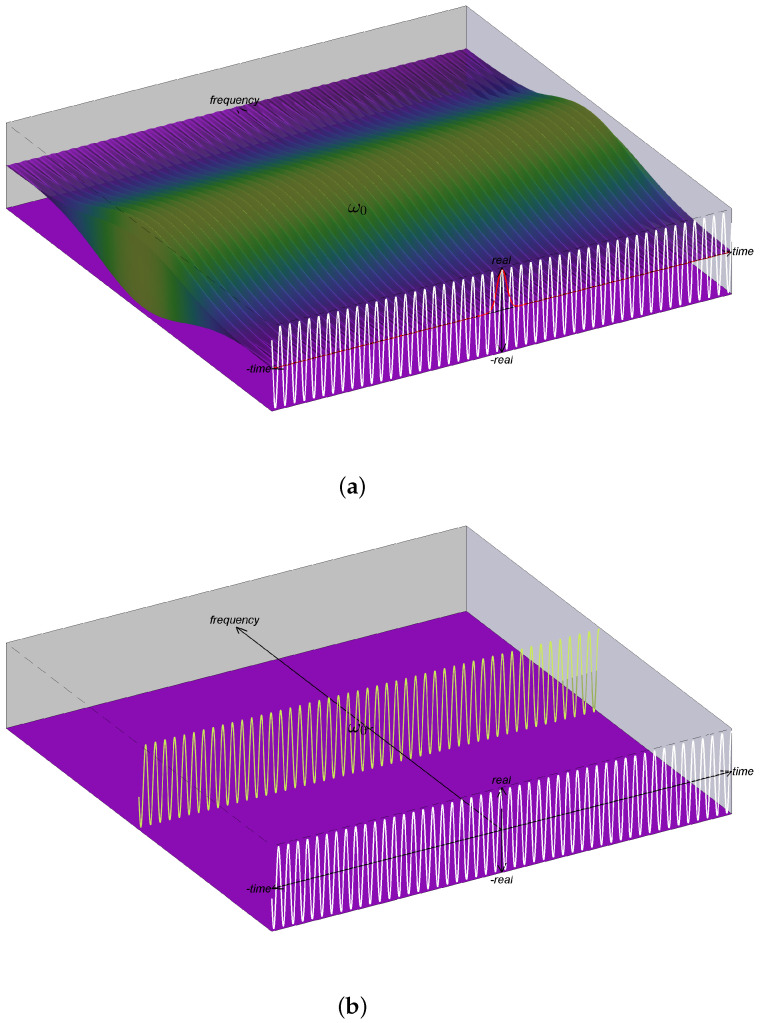
Illustrations associated with the filterbank interpretation of the STFT for a complex exponential. For the complex exponential, (**a**) shows a visualization of $Z_{w}\left(t,\nu \right)$ where the coloring is based on magnitude and height reflects the real value; *this plot is not a valid IS*. The plot in (**b**) shows $S^{\mathrm{FB}}\left(t,\omega \right)$, which is the correct IS after reassignment.

The FB interpretation of the STFT in (35) is shown in Figure 2a. As discussed in Section 5.2, this STFT is not a valid IS and the structural problem may be seen in (36) and observed in the figure. In particular, the IF of the component corresponding to the channelizer with center frequency ν is given by $\frac{\;d}{\;dt}\theta_{w}\left(t;\nu \right)=\omega_{0}$, i.e., the IF has constant value $\omega_{0}$ and is thus independent of the value ν. This can be seen in the figure by observing that the oscillation rate of components is fixed and does not change along the frequency axis. On the other hand, reassigning this IS using (24) gives (37), which is illustrated in Figure 2b.

### 6.2. Linear FM Chirp

In the second example, consider the canonical triplet

(38) $$C_{0}=\left(1,\;\;\omega_{0}+\omega_{c}t,\;\;0\right)$$

which using (16a) gives the linear FM chirp signal

(39) $$z\left(t\right)=\exp\left[j(\omega_{0}t+\frac{\omega_{c}}{2}t^{2})\right]$$

and with (14) gives the IS

(40) S(t,ω)=2πδω−(ω0+ωct)00ej(ω0t+ωc2t2).

Choosing the Gaussian window in (32), we compute the STFT corresponding to the FT interpretation by using (5); choosing $p_{1}=-j\omega_{c}/2$, $p_{2}=\beta^{2}/2$, $t_{1}=0$, and $t_{2}=\tau$ in (28) and using time- and frequency-shift properties of the FT

(41) $$\begin{array}{c} Z_{w}\left(\omega ;\tau \right)=\\ |\lambda |\exp\left[\frac{\beta^{2}\left(\beta^{4}-\gamma \right)\tau^{2}+\beta^{2}\omega_{c}\tau \left(\omega -\omega_{0}\right)-\beta^{2}{\left(\omega -\omega_{0}\right)}^{2}}{2\gamma }\right]\\ \times \exp\left[j\frac{\beta^{4}\omega_{c}\tau^{2}-2\beta^{4}\tau \left(\omega -\omega_{0}\right)-\omega_{c}{\left(\omega -\omega_{0}\right)}^{2}}{2\gamma }+j\arg\left\{\lambda \right\}\right] \end{array}$$

where $\lambda =\beta /\sqrt{\beta^{2}-j\omega_{c}}$. The IS corresponding to the FT interpretation $S_{\tau }^{\mathrm{FT}}\left(t,\omega \right)$ is then formed by substituting the above into (19).

Next, we compute the STFT corresponding to the FB interpretation using (41) and (10), then, in polar form (see (23)) we have

$$\begin{array}{ccc} a_{w}\left(t;\nu \right) & = & |\lambda |\exp\;\left[\frac{\left(\beta^{4}-\gamma \right)t^{2}+\omega_{c}t\left(\nu -\omega_{0}\right)-{\left(\nu -\omega_{0}\right)}^{2}}{2\gamma \beta^{-2}}\right] \end{array}$$

and

$$\begin{array}{ccc} \theta_{w}\left(t;\nu \right) & = & \frac{\beta^{4}\omega_{c}}{2\gamma }t^{2}-\frac{\beta^{4}\left(\nu -\omega_{0}\right)}{\gamma }t-\frac{\omega_{c}{\left(\nu -\omega_{0}\right)}^{2}}{2\gamma }+\nu t+\arg\left\{\lambda \right\}. \end{array}$$

Finally, the IF as a function of ν is given by

$$\begin{array}{ccc} \frac{\;d}{\;dt}\theta_{w}\left(t;\nu \right) & = & \frac{\beta^{4}\omega_{c}}{\gamma }t-\frac{\beta^{4}\left(\nu -\omega_{0}\right)}{\gamma }+\nu . \end{array}$$

The IS corresponding to the FB interpretation of the STFT $S^{\mathrm{FB}}\left(t,\omega \right)$ is readily obtained from the equations above together with (24).

As in the previous example, $S_{\tau }^{\mathrm{FT}}\left(t,\omega \right)$ does not allow direct comparison with (40) because it yields a continuum of ISs. Thus, superposition of the IS continuum across τ would only lead to $S^{\mathrm{FD}}\left(t,\omega \right)$. However, unlike in the previous example, reassignment of $Z_{w}\left(t;\nu \right)$ to form $S^{\mathrm{FB}}\left(t,\omega \right)$ does not yield the correct IS given in (40). Although reassignment improves energy concentration in time-frequency representations, it is unlikely to lead to the correct IS in general. We note that other variations of syncrosqueezing methods (e.g., higher order and adaptive methods) exist [18,19,30,31,32] that may perform well for specific signals, but in general there exists no single method that is well suited for all signals.

The ISs corresponding to the FT interpretation of the STFT in (41) are shown in the right column of Figure 1. The top plot shows $S^{\mathrm{FD}}\left(t,\omega \right)$ for the linear FM chirp while the lower three plots show $S_{\tau }^{\mathrm{FT}}\left(t,\omega \right)$ for three different τ [see (19)]. As before, it is apparent that the frequency spectrum which results from taking the FT of any window grain has components that extend beyond the time support of the window, further demonstrating the conceptual flaw in the FT interpretation of the STFT.

**Figure 3:**
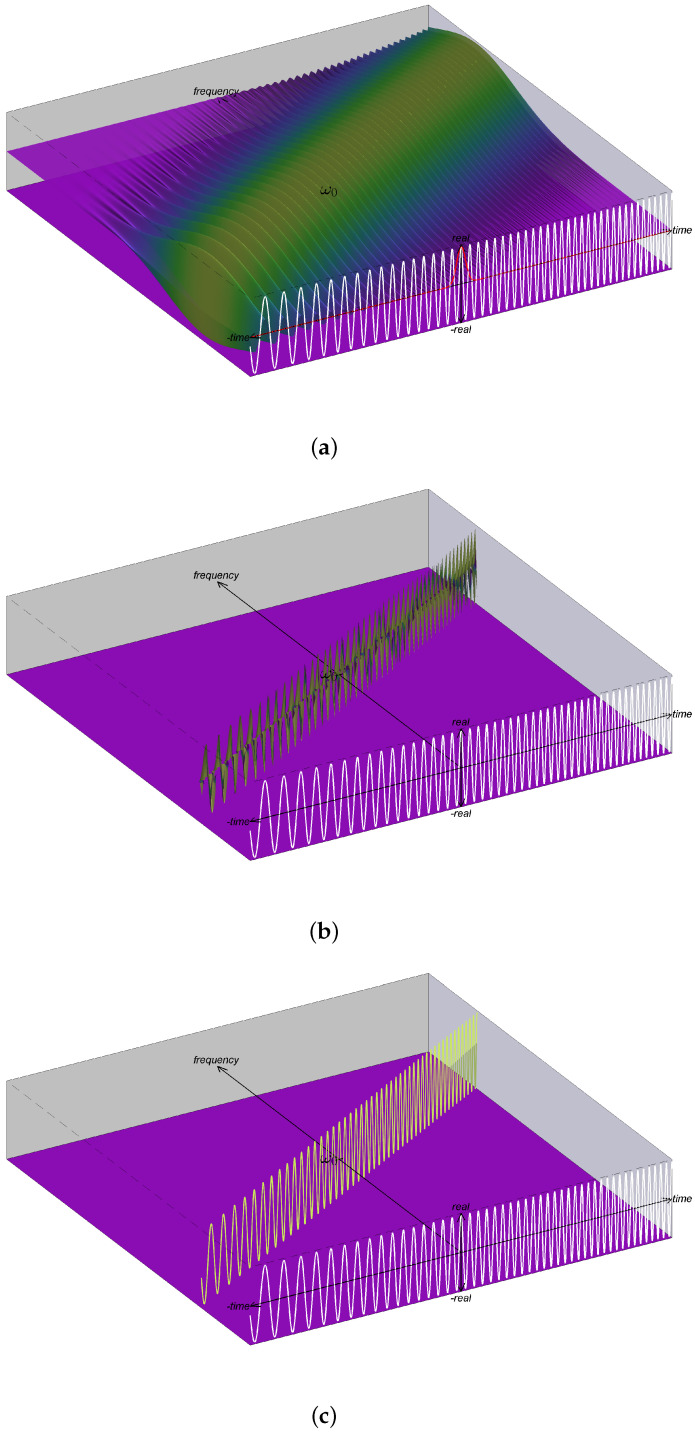
Illustrations associated with the filterbank interpretation of the STFT for a linear FM chirp. For the linear FM chirp, (**a**) shows a visualization of $Z_{w}\left(t,\nu \right)$ where the coloring is based on magnitude and height reflects the real value; *this plot is not a valid IS*. The plot in (**b**) shows $S^{\mathrm{FB}}\left(t,\omega \right)$, which shows that reassignment improves energy concentration, but does not lead to the correct IS [shown in (**c**)] as explained in Section 6.

The FB interpretation of the STFT in (41) is shown in Figure 3a. Again, we see in the figure that the oscillation rate of components is fixed and does not change along the frequency axis. With reassignment, the resulting IS shown in Figure 3b [15] has improved energy concentration but does not give the correct IS provided in (40) and shown in Figure 3c.

### 6.3. Bat Vocalization

Finally, we illustrate the recasting of a synchrosqueezed STFT as an IS (i.e., the IS corresponding to the FB interpretation of the STFT) using a bat vocalization signal which is popular in the time-frequency literature [18,33,34,35]. The acoustic recording features a ∼2.5 ms pulse emitted by the Large Brown Bat *Eptesicus Fuscus*. The original recording consists of 400 samples captured with a sampling period of 7 μs. In order to alleviate issues associated with numerical derivatives when recasting as an IS, we up-sampled the signal by 4×. Finally, a 128 point Hann window was used in the analysis.

**Figure 4:**
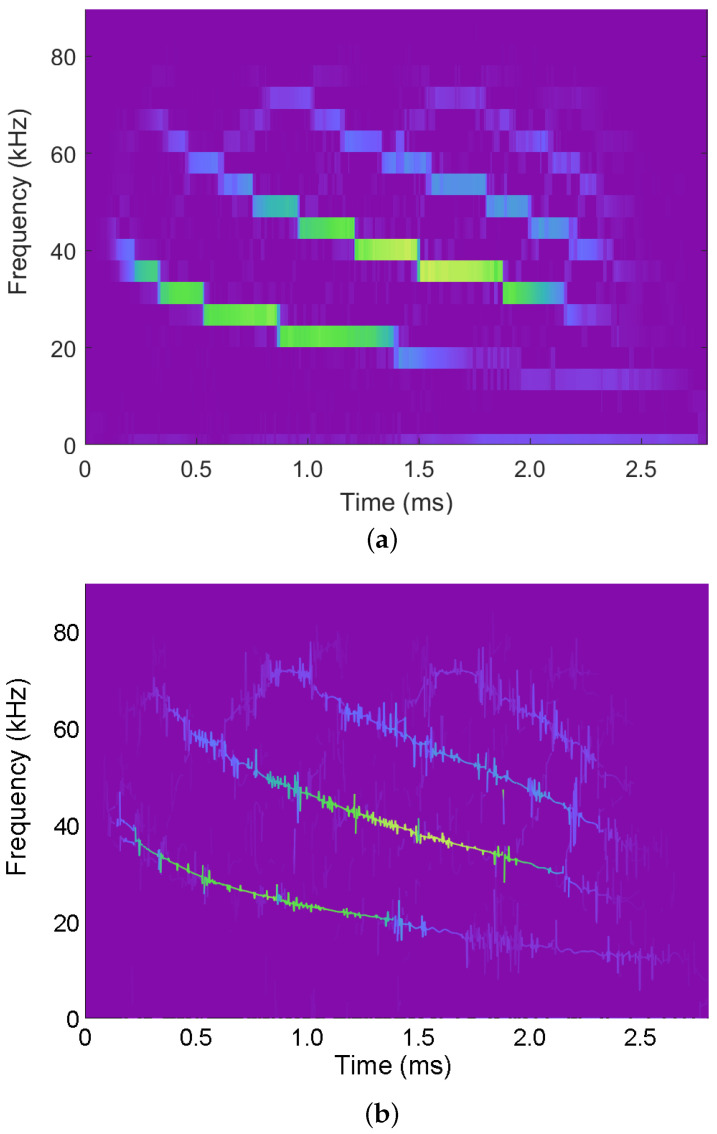

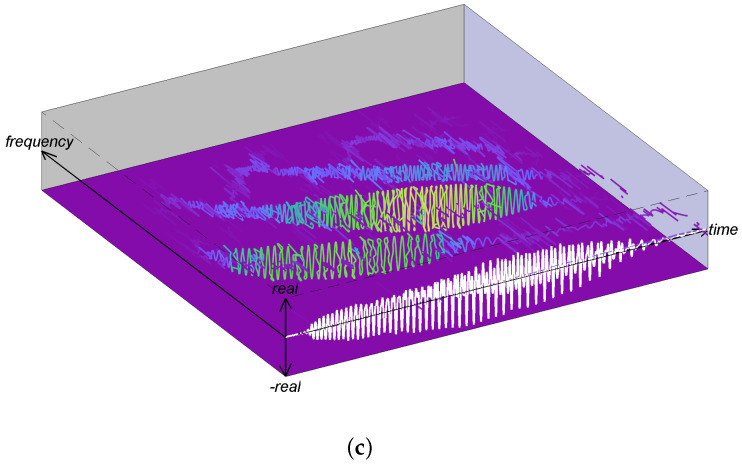
Illustrations associated with an acoustic bat vocalization recording for the (**a**) synchrosqueezed STFT, (**b**) 2D IS obtained by recasting the FB interpretation of the STFT, and (**c**) 3D IS. Broadly speaking, the energy in the subplots are in general agreement, however, the IS provides more precision that allows the display of finer details and the 3D IS allows the illustration of the spectral phase.

The synchrosqueezed STFT, shown in Figure 4a, was computed using the fsst() function in Matlab and plotted using the default Matlab visualization (with a perceptual colormap). For comparison, the 2D IS corresponding to the FB interpretation of the STFT is shown in Figure 4b. Finally, the 3D IS corresponding to the FB interpretation of the STFT is shown in Figure 4c. Broadly speaking, the energy in Figure 4a,b are in general agreement, however, the IS provides more precision that allows the display of finer details. Moreover, while both Figure 4a,b provide information about the magnitude in the time-frequency plane, by leveraging the 3D IS visualization, we are able to additionally illustrate the spectral phase.

## 7. Conclusions

In this paper, we used the ISA framework to recast the FT and FB interpretations of STFT in terms of an IS. We showed that these two equivalent STFT interpretations lead to different ISs, and thus provide new insights into STFT: the FT interpretation of STFT corresponds to an IS for each window grain, while the FB interpretation of STFT is a valid IS if the STFT is synchrosqueezed. Thus, we provided a new theoretical motivation for synchrosqueezing, which is a fundamental necessity in order to cast the FB interpretation of STFT as a valid IS. We also highlighted the differences in the components for these interpretations, which have significant conceptual and practical differences. Specifically, in the FT interpretation, the components have a restrictive fixed amplitude and fixed frequency, while in the FB interpretation the components are AM–FM in nature. We leveraged the 3D IS visualization to provide a novel visualization of an STFT in which multiple aspects, i.e., magnitude and phase of each signal component, can be viewed simultaneously. Moreover, the phase is visualized in a way that is easily interpreted—this is in stark contrast with typical STFT analysis where phase is rarely visualized because it is not easily interpreted. Finally, in order to demonstrate these relations and results, we provided examples and illustrations.

## Notation

The following notation is used throughout this work:

z(t)	signal under analysis
w(t)	window signal
$Z_{w}\left(\omega ;\tau \right)$	classical STFT corresponding to the FT interpretation
$Z_{w}\left(t;\nu \right)$	modified STFT corresponding to the FB interpretation
$Z_{w}\left(t;\nu \right)e^{-j\nu t}$	classical STFT corresponding to the FB interpretation
S(t,ω)	an IS corresponding to the signal z(t)
$S_{\tau }^{\mathrm{FT}}\left(t,\omega \right)$	IS corresponding to the FT interpretation of the STFT
$S^{\mathrm{FB}}\left(t,\omega \right)$	IS corresponding to the FB interpretation of the STFT
$S^{\mathrm{FD}}\left(t,\omega \right)$	IS corresponding to the frequency domain (FT)
